# A Local 3D Voronoi-Based Optimization Method for Sensor Network Deployment in Complex Indoor Environments

**DOI:** 10.3390/s21238011

**Published:** 2021-11-30

**Authors:** Ali Afghantoloee, Mir Abolfazl Mostafavi

**Affiliations:** 1 Center for Research in Geospatial Data and Intelligence, Laval University, Quebec City, QC G1V 0A6, Canada; mir-abolfazl.mostafavi@scg.ulaval.ca; 2 Center for Interdisciplinary Research in Rehabilitation and Social Integration, Laval University, Quebec City, QC G1V 0A6, Canada

**Keywords:** deployment, sensor network, indoor, 3D environment, 3D Voronoi structure

## Abstract

Optimal sensor network deployment in built environments for tracking, surveillance, and monitoring of dynamic phenomena is one of the most challenging issues in sensor network design and applications (e.g., people movement). Most of the current methods for sensor network deployment and optimization are empirical and they often result in important coverage gaps in the monitored areas. To overcome these limitations, several optimization methods have been proposed in the recent years. However, most of these methods oversimplify the environment and do not consider the complexity of 3D architectural nature of the built environments specially for indoor applications (e.g., indoor navigation, evacuation, etc.). In this paper, we propose a novel local optimization algorithm based on a 3D Voronoi diagram, which allows a clear definition of the proximity relations between sensors in 3D indoor environments. This proposed structure is integrated with an IndoorGML model to efficiently manage indoor environment components and their relations as well as the sensors in the network. To evaluate the proposed method, we compared our results with the Genetic Algorithm (GA) and the Covariance Matrix Adaptation Evolution Strategy (CMA-ES) algorithms. The results show that the proposed method achieved 98.86% coverage which is comparable to GA and CMA-ES algorithms, while also being about six times more efficient.

## 1. Introduction

Advances in sensor technologies increasingly allow efficient and on-the-fly tracking and monitoring of diverse dynamic phenomena for different applications. These applications may include disaster management, traffic management, security surveillance, flood monitoring, and real-time tracking for mobility applications [1]. For the efficient tracking and monitoring of dynamic phenomena using a sensor network, different issues related to sensor types, sensing models, connectivity, communication, location and coverage as well as efficient assessment of real-time measurements need to be addressed.

Among these issues, optimal sensor placement is a prerequisite for efficient monitoring and coverage in a given environment. Traditional methods for sensor network deployment are mostly experimental and not efficient. Other optimization approaches are rarely context-aware and they do not consider the complexity of the environment [2,3,4,5]. In addition, these methods usually oversimplify sensor models (sensing fields). This is especially true for the built indoor and outdoor environments where the presence of diverse features such as buildings, walls, trees, floors, and stairs complexify not only the communication between sensors but also their placement and optimization. In recent years, there have been some efforts to improve sensor models and hence increase the quality of measurements obtained from a sensor network. For instance, in [6], the deployment of wireless cameras has been carried out by considering the quality of images related to light resources, distortion phenomena in cameras, and coverage area, as well as a constraint regarding connectivity between cameras in indoor environments. However, several problems regarding the optimal deployment of sensor networks in indoor environments remain. In fact, the existing optimization methods for sensor network deployment and optimization do not consider the complexity of 3D environments and their architectural design. It is also very challenging to consider the presence of diverse obstacles in such environments in the optimization process.

Most of the existing research works for sensor network optimization are based on stochastic approaches which are generally very time-consuming and are not efficient. In contrast to stochastic approaches, approaches based on local optimization methods, Virtual Force [2] and Voronoi diagram [3], have demonstrated a better performance in this regard. Virtual force approaches have a high number of parameters allowing the algorithm to stick in the local optimums, whereas the Voronoi diagram is a spatial partitioning method that helps to efficiently manage the proximity information between objects (sensors) with minimum setting parameters [7]. Despite the aforementioned advantages, there have been few works [4,8,9] based on these approaches for sensor placement in a 3D indoor environment.

Hence, in this paper, we propose a 3D Voronoi-based optimization method for sensor network (e.g., omnidirectional cameras) deployment in 3D complex indoor environments. The proposed method benefits from the combination of a 3D Voronoi diagram with the 3D vector environment model (IndoorGML) to efficiently manage both the indoor environment and its components as well as the spatial distribution and relations between sensors in the network. To illustrate the validity of the proposed method, three experiments were conducted. The first experiment was designed to evaluate the efficiency of our proposed algorithm for the placement of four omnidirectional cameras with unlimited sensing ranges. Then, the sensitivity of the proposed method, with respect to limited sensing range and the number of cameras, was investigated in the second and third experiments.

The remainder of this paper is organized as follows. In Section 2, recent studies on sensor networks deployment issues are reviewed. Next, in Section 3 and Section 4, we present our optimization methods for sensor network deployment in an indoor environment that considers both the outer (environmental) and inner (sensor) factors. Then, to illustrate the strengths of the proposed 3D Voronoi-based approach for the deployment of a sensor network in an indoor environment, a case study carried out in the Quebec City Convention Center is presented in Section 5. Finally, in Section 6, the results are compared with two stochastic optimization methods (Genetic algorithm (GA) and Covariance Matrix Adaptation Evolution Strategy (CMA-ES)) and conclusions and future works are stated.

## 2. Related Works

Optimal deployment of a sensor network to enable an indoor environment for the tracking and monitoring of its dynamics for applications such as mobility and navigation activities is a challenging task [10,11]. Coverage problems in sensor networks have been intensively studied in the last decade. Depending on the type of application, we may be interested in the target or area-based coverage. This implies that in some sensor network applications, monitoring target points such as buildings, doors, and corridors are desired, while in other applications, the aim is the detection of mobile targets such as intruders [11]. Hence, covering the maximum number of target points by some level of certainty, instead of the whole area, is the main objective in the target-based coverage problems [12]. However, the presence of obstacles may complicate monitoring tasks and the achievement of such a goal [13]. To overcome these limitations, some efforts have been made based on visibility graphs by considering the presence of obstacles to compute the best coverage path between sensors and targets [14].

In the area-based coverage problem, which is the focus of this paper, the objective is to maximize the area covered by sensors. The quality of the coverage is expressed by the ratio of the covered area to the whole area [5]. The area-based coverage calculation methods are classified into: (1) raster-based methods [4,8,9], which are limited by the spatial resolution; and (2) the vector-based models [3,11,15,16,17]. These methods have been mostly applied for 2D spaces and rarely consider the presence of man-made objects in the environment.

In recent years, several methods (e.g., global or local, deterministic or stochastic) have been proposed to optimize the configuration of sensors in a sensor network based on the maximum coverage criteria [18]. Genetic Algorithm (GA) [19], Particle Swarm Optimization (PSO) [20], Simulated Annealing (SA) [21], and Covariance Matrix Adaptation Evolution Strategy (CMA-ES) [18] are examples of global optimization methods. In these methods, an objective function for the whole network reconfiguration is used, whereas in local algorithms such as Virtual force-based [2], VECtor-based, and Voronoi diagram-based algorithms [3], reconfiguration of sensors is locally done with respect to the presence of neighboring sensors and the local environmental context.

Global algorithms have been used for globally optimizing the configuration of sensor networks. In these algorithms, for each optimization step, the cost function for each solution must be estimated for the computation of the next generation of solutions. For instance, in the GA algorithm, the cost function must be calculated for 100 solutions in each iteration, if the number of iterations is 100, the number of cost function calculation is 10,000. If the time of cost function calculation is 3 s, the total time becomes 30,000 s. Hence, global algorithms are highly time-consuming due to the high number of cost function (e.g., coverage) computations for sensor network optimization.

In contrast, in most of the local optimization algorithms, no cost function is used to define the movement strategy of sensors within a network [22]. Indeed, these algorithms attempt to maximize the network coverage according to the local spatial information on the environment as well as the information related to the configuration of the neighboring sensors. For instance, in Yaagoubi et al. [23], a Voronoi-based algorithm is used for the deployment of a set of cameras by considering the information on the structure of the environment. The results show that the optimal places for cameras are close to the edges of Voronoi cells. In general, although these algorithms are fast and do not need to recalculate the cost function as in global approaches, they have limitations in terms of the consideration of constraints (e.g., none-deployable spaces), consideration of multi-objective optimization goals, and complex sensing models of the sensors.

One of the key issues of all sensor deployment optimization algorithms is the accurate estimation of an individual sensor coverage in a sensor network. The sensing model of a sensor may be binary or probabilistic, omnidirectional, or directional. Precise sensing model consideration has a significant impact on the quality of coverage estimation of a sensor network. Most of the sensor coverage estimation methods use a raster representation of the environment for optimization purposes [8], which limits their precision and efficiency. This is because raster representations are constrained by their spatial resolution, and their regular shapes resulting in redundant data for unoccupied areas (i.e., the unoccupied pixels are considered in the storage volume of raster data). Moreover, raster-based 2D models cannot be used for indoor environments to represent solid objects and columns. To overcome these limitations, in [24] a sensor coverage estimation method has been proposed based on the precise 3D vector-based representation of the environment, which can be used for the estimation of the sensor network coverage in an indoor environment.

In addition to raster and vector representation, the voxel representation of the environment has been proposed for the deployment of a camera network to track a 3D environment [25]. Each voxel value shows the presence or absence of an object in the space. The maximum visibility of cameras was achieved by a cooperative algorithm which splits the problem to several sub-problems [26]. Although the voxel representation has the potential of modeling solid objects and is appropriate for indoor environments, the visibility estimation is sensitive to the resolution of the voxels.

Concerning indoor versus outdoor environments in the sensor deployment problem, the majority of research works have been developed for outdoor environments, and a few research works have considered indoor environments for sensor deployment [6]. For instance, in Konda and Conci’s [6], a set of cameras were deployed using the Genetic Algorithm as a global optimization algorithm to achieve the maximum coverage with high-quality image output by considering the light resource and obstacles in the indoor environment. Furthermore, a greedy sensor deployment optimization on the ceilings of a building (in a simulation case) was conducted for monitoring the indoor area [27]. In this study, some virtual obstacles were considered in the simulation and the floor surfaces were chosen as the coverage area.

In summary, most of these methods oversimplify the sensing models for each sensor (circle or spherical in the best case), as well as the environment (2D, without obstacles), for achieving the maximization of coverage. Additionally, the majority of these works were developed for outdoor environments, and only a few research works have considered indoor environments for sensor deployment [6]. To address these challenges, we propose a 3D Voronoi-based optimization method for sensor network deployment in 3D complex indoor environments. This method is presented in the following sections.

## 3. Methodology

In recent years, several strategies have been developed for the local optimization of sensor networks based on the Voronoi structure, which has demonstrated its potential especially for the efficient management of neighborhood information in sensor networks. For example, Argany et al. [22] proposed a local optimization algorithm based on a 2D Voronoi diagram where each sensor was moved towards the farthest vertex of its cell iteratively to fill coverage holes in a sensor network in an outdoor environment. Doodman et al. [28] proposed a Voronoi diagram-based approach to define movement strategy for sensors by keeping them away from obstacles in the environment. These works had the advantage of performing the optimization process more efficiently compared to global optimization algorithms.

**Figure 1:**
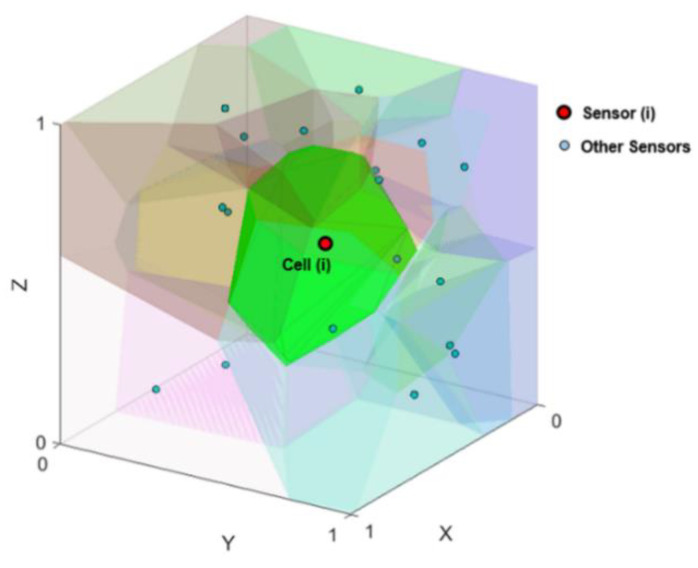
Voronoi diagram for sensor networks in 3D space.

Given these advantages, here we propose a local context-aware optimization algorithm based on 3D Voronoi diagrams for the deployment of a sensor network in an indoor environment. In the proposed method, a 3D Voronoi diagram is used for the representation of sensors positions and their topological relations within a sensor network distributed in an indoor environment. A 3D Voronoi diagram also provides the necessary spatial structure for the manipulation and management of the sensors’ positions and their movement for optimization purposes. A 3D Voronoi diagram, for a set of points, partitions a 3D space into polyhedral convex volumes called 3D Voronoi cells (Figure 1).

A 3D Voronoi cell is calculated based upon the following definition:

(1) $$VC_{i}=\{p_{i}\;\in \;R:d\left(p_{i},S_{i}\right)<d\left(p_{i},S_{n}\right)\}$$

where, $S_{i}$ and $S_{n}$ are the positions of sensor *i* and any other sensor *n*, respectively. $VC_{i}$ is a 3D Voronoi cell, $p_{i}\;$is any point inside a $VC_{i}$ in a 3D space *R*, and *d* is the distance between $p_{i}$ and any sensor $S_{i}$.

**Figure 2:**
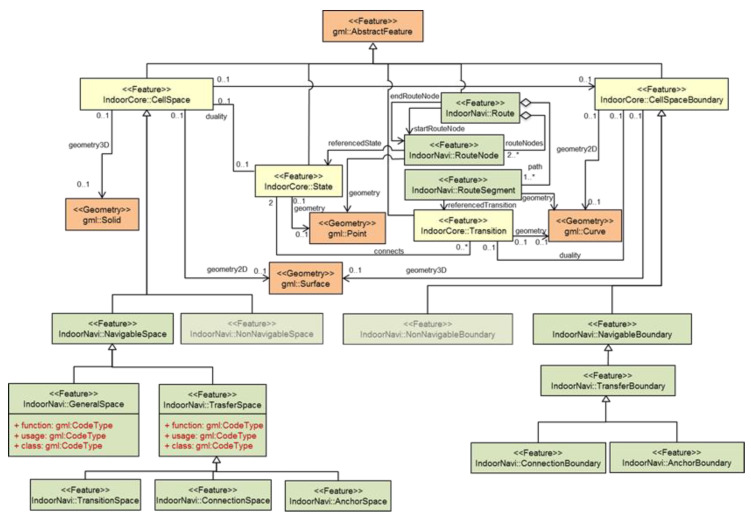
UML diagram of the IndoorGML core module and the navigation module (OGC, 2014).

For integrating context information from the 3D indoor environment in the optimization process for a sensor network represented with a 3D Voronoi diagram, we considered benefiting from the potential of the 3D IndoorGML data model proposed by the OGC. 3D IndoorGML is an extension of CityGML (level of detail (LoD) 4) that provides semantic, topological, and spatial information of objects and services [29]. IndoorGML is an open standardized data model of the interior space of 3D buildings and is composed of a core module, an appearance module, and a thematic module [30]. The main structure of IndoorGML divides an indoor space into multi-spaces called cells. The intersection area of two neighboring cells is called a boundary surface [30]. IndoorGML uses two dual spaces to model indoor environments: (1) primal space, the geometric representation of a cell space and a cell boundary space; and (2) dual space, the Node Relationship Graph representation of cells and their boundary surfaces, which respectively correspond to nodes and edges (Figure 2). IndoorGML is extended with the navigation core module that has two classes, namely, navigable space and non-navigable space. The non-navigable space represents the cells occupied by obstacles and walls. The navigable space includes all the connection spaces (e.g., doors), anchor spaces (e.g., building exits), general spaces (e.g., rooms), and transition spaces (e.g., corridors). In contrast, CityGML includes boundary surfaces, rooms, openings, and closure surfaces (e.g., the space between the kitchen and the living room is a virtual surface called closure surface) [31]. IndoorGML has an external reference that enables engines and data interpreters to link with CityGML to access the semantic of the surfaces.

**Figure 3:**
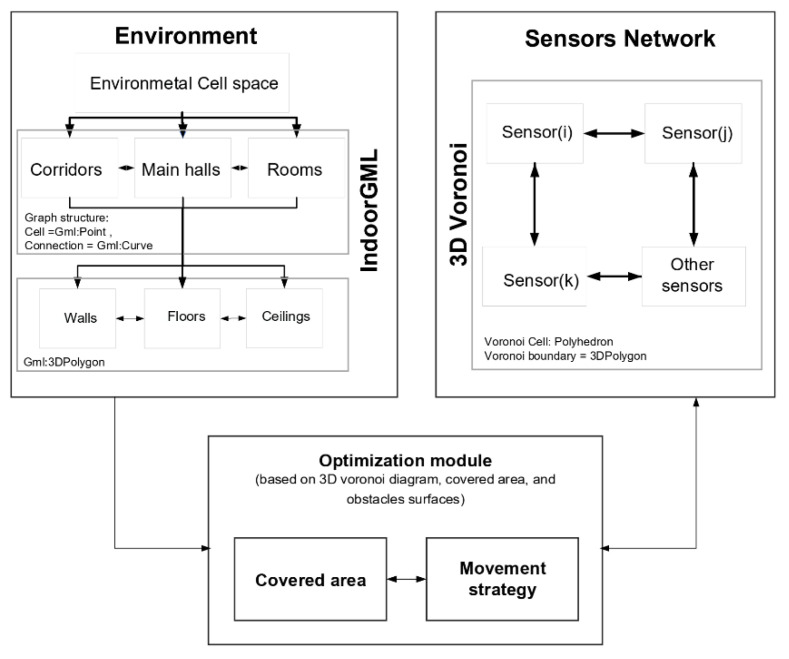
The proposed sensors deployment approach in a 3D indoor environment based on IndoorGML and a 3D Voronoi diagram.

Hence, we propose an integrated model for optimal sensor placement in an indoor environment that combines IndoorGML classes to represent indoor environments and a 3D Voronoi diagram for the representation of sensor positions and their proximity relations in 3D indoor spaces. Hence, our proposed model is composed of three main components including (1) the environment, (2) the sensor network, and (3) the optimization module (Figure 3). The optimization algorithm uses the information of the indoor environment represented by IndoorGML and the neighborhood relations of sensors defined by a 3D Voronoi diagram. The optimization module includes our iterative local optimization algorithm. The information provided by the first two components will allow us to estimate, at each iteration, the total coverage provided by the sensor network and decide on the changes in the network in subsequent steps (i.e., individual sensor movement) to improve the network total coverage value.

In the proposed integrated model, 3D Voronoi structure uses the information represented by IndoorGML to guide the optimization process. IndoorGML defines the indoor environment structures with a set of cells and store them with their central points as nodes and with links to define the cells connections. In addition, each cell is stored using a surface geometry. This structure is used as the sensor deployable space. We also add semantic information to the cells and surfaces described by the IndoorGML model to better guide sensor placement. We use the 3D Voronoi structure together with this information to manage sensors movement and the updated proximity information between sensors. The cells in the IndoorGML model are divided into three main classes according to the priority of the sensor deployment places for mobility activities. These include: (1) corridors; (2) main halls; and (3) rooms. Surfaces are also classified for each cell into three classes: ceilings; walls; floors; and obstacles. To calculate a sensor network coverage, individual sensor position and coverage must be determined using the information provided through the IndoorGML model.

**Figure 4:**
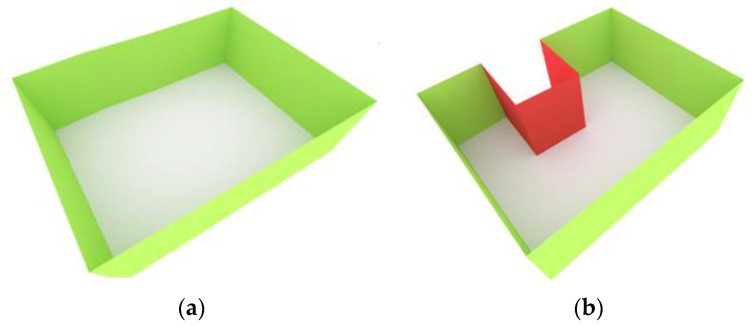
(**a**) A convex cell represented by its walls surfaces (green) and its floor surface (white). (**b**) A non-convex cell that includes several wall surfaces as obstacles (red).

In our study, the floor surface of each cell is aimed to be covered by the sensor network. According to the sensors’ positions in the Voronoi structure and considering the presence of various obstacles in the environment, the coverage estimation is calculated using a visibility approach [24]. In this regard, the obstacles are defined as objects that obstruct the sensor’s vision. For instance, Figure 4 shows the red surfaces as obstacles that reduce the covered area of sensors in a non-convex area. Sensor deployable spaces are composed of the walls and ceilings of each cell space.

As mentioned, the sensor network optimization algorithm is included in the optimization module. The iterative algorithm is developed for the maximization of the sensor network coverage. In each iteration, an individual sensor is moved towards its new position that aims at improving the total sensor network coverage. This movement is based on a context-aware approach and is sensitive to the shape of the environment, the presence of obstacles in the environment as well as the presence of other sensors in the vicinity of the sensor being assessed.

**Figure 5:**
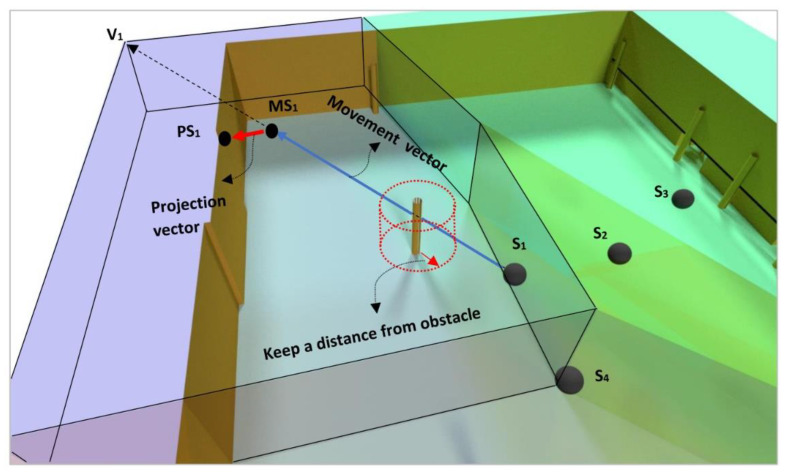
Movement strategy of sensors inside a 3D space based on a 3D Voronoi diagram (colored 3D cells) with consideration of projection and obstacle avoidance.

As a movement strategy, each sensor is moved towards the farthest vertex of its 3D Voronoi cell by adding a repulsive force to keep the sensor away from obstacles and keep its position on the wall or ceiling. This strategy mainly allows for reducing the overlapping coverage to maximize the coverage of the target areas. For example, ${\vec{V}}_{s_{1},v1}$ in Figure 5 illustrates the direction of the movement of S_1_ allowing its displacement from other sensors. In Figure 5, Sensor 1 (S_1_) is moved to position MS_1_ in the direction of ${\vec{V}}_{s_{1},v1}$, and is then projected on the nearest plane (position PS_1_). In the strategy, the sensor should be kept away from the obstacle’s surfaces based on a defined distance.

## 4. Formal Representation of the Proposed Optimization Algorithm

In this section, we formally present the pseudo-code of our proposed local optimization method based on a 3D Voronoi diagram for sensor network deployment in complex indoor environments (Algorithm 1). In this algorithm, we assume a network of omnidirectional cameras for the coverage of the indoor environment. The objective of sensor deployment is to maximize the covered areas to support applications such as indoor navigation or security purposes. We also assume that sensors can be deployed mainly on the walls and ceilings. Building floors are considered as target areas to be covered where people’s activities are expected. In addition to sensor characteristics and neighborhoods, information from the 3D indoor environment is used as context information for optimal sensor network deployment. The presence of other objects embedded in the indoor environment that may affect coverage information is also considered (e.g., presence of a column or other permanent obstacles like walls in the environment).

Thus, in the proposed algorithm, first, we create a 3D Voronoi diagram using a set of generating point $\left(\mathrm{sensors}\;S_{1},\ldots ,\;S_{n}\right).$ The 3D Voronoi diagram is obtained from its dual Delaunay tetrahedralization (DT) which is calculated based on an incremental construction approach [32]. The incremental method for the construction of a Delaunay tetrahedralization consists of three main steps including initialization, point insertion and the tetrahedralization optimization. The process starts with the definition of a universal tetrahedron large enough to enclose all the generating points (sensors) in the 3D space. Then, sensors are inserted one by one in the existing tetrahedralization using a search function to find the tetrahedron that contains the newly inserted point. Then, the tetrahedralization is locally updated to make sure that all the tetrahedrons respect Delaunay criterion which requires that the circumsphere of each tetrahedron is empty. Indeed, the circumcentres of the final tetrahedrons are dually considered as the vertices of 3D Voronoi polyhedrons. Hence, 3D Voronoi cells are generated by connecting all the circumcentres of the neighboring tetrahedrons that share a given generating point.

**Figure 6:**
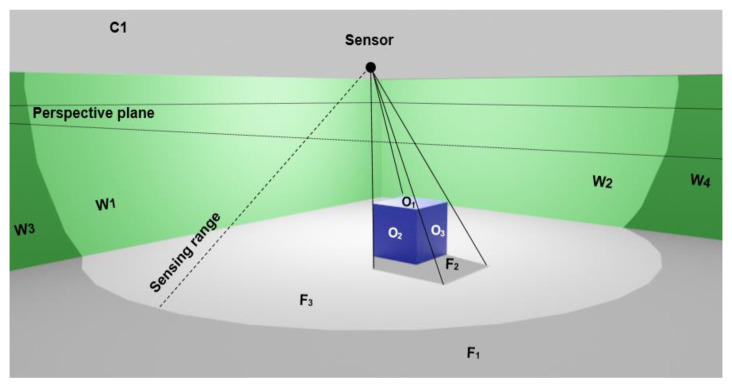
Coverage estimation based on overlapping visible surfaces projected onto the perspective plane. Surface F_1_ is out of whole area of sensing distance, and surface F_2_ is hidden by surface O_1_.

In the next step, we initiate current coverage value [24] for each sensor in the network. The coverage value of each sensor in a 3D space is estimated based on the method proposed in following steps [24]. In this method, first, we eliminate the irrelevant surfaces for the computation of the coverage of each sensor. These include: (1) elimination of the back-face surfaces (e.g., surface O_2_ and O_3_ in Figure 6) in which the angle between the normal vector of a back-face surface and the sensor direction is less than 90 degrees, (2) elimination of the surfaces that lie on the back side of sensor deployable surface (especially for the walls), and (3) elimination of the surfaces that lie outside the sensing distance (e.g., surface F_1_). Next, we project the remaining surfaces on a perspective plane which is defined parallel to the floor surface. Then, we overlay these surfaces in the projection plane (e.g., projected surface of O_1_ = F_2_ and F), in order to find the visible part of the floor (surface F-F_2_ = F_3_) covered by the sensor. Finally, we calculate the covered area by the given sensor on the floor (F_3_).
**Algorithm 1.** Pseudo-code of the 3D Voronoi approach for sensor network optimization in an indoor environment input: *n* omnidirectional cameras *Si*(*x_i_*, *y_i_*, *z_i_*)
output: (*X_i_, Y_i_, Z_i_*) optimal solution objective: Maximizing the coverage of the camera network1**Initialize**: Random distribution of the cameras on deployment planes2(walls/ceilings) Compute initial sensor network coverage;33D_Voronoi(*S_i_,...,S_n_*);4step*_*size ← initial*_*step*_*size;5**for***i* ← 1 **to**
*n*
**do**6 *S′_i_ ← Movement strategy(S_i_);*7 {8    1- choose the farthest vertex in the same direction of path segments;9    2- amount of movement is *step*_*size* * vector’s length;10    3- project the movement vector on the nearest sensor deployed plane;11    4- if the movement vector has an intersection with an obstacle, keep a given distance12    between sensor and obstacle;13 }14 *g’S_i_ ← coverage (S_1_,...,S’_i_,...,S_n_)-coverage (S_1_,...,S_i_,...,S_n_);*15 *PQ ← add(g’S_i_,S’_i_)*;16**end**17*PQ ← Sort(PQ, highest gain)*;18*K ← 1;*19**while** (not terminated condition) **do**20 *step_size ← (initial_step_size) ∗ (Iteration − K)/Iteration;*21 *S’u ←PQ*(1)*.S;*22 *Su ←S’u;*23 *Update_3D_Voronoi(S_i_,...,S_u_...,S_n_)*;24 *S’_u_ ← Movement strategy(S_u_);*25 g’*S’_u_ ← coverage (S_1_,...,S’_u_,...,S_n_)-coverage (S_1_,...,S_u_,...,S_n_);*26 *PQ ← add(g’S’_u_,S’_u_);*27 **for***j* ← 1 **to**
*N_neighboringSu*
**do**28  *NS’_i_ ← Movement strategy(NS_j_);*29  *g’NS_j_ ← coverage (S_1_,...,NS’_j_,...,S_n_)-coverage (S_1_,...,NS_j_,...,S_n_);*30  *PQ ← add(g’NS_j_,NS’_j_);*31 **end**32 *PQ ←Sort(PQ, highest gain);*33 *K ←K* + 1;34**end**

Next, we establish a priority queue (*PQ*) for the management of sensor movement [22]. To do so, we compute the floor surfaces as coverage gain value for each sensor based on their first movement. The value and orientation of this movement is defined based on the information on local context as well as the sensor network configuration. Then, the sensor movements are organized from maximum to minimum order according to the coverage gain value (*g_si_*) they produce. Hence, the first sensor in the list will have the priority to move before the others.

As mentioned, the sensor movement strategy is the core element of the proposed algorithm. The strategy for a sensor *i* (*S_i_*) to move to its new position (*S′_i_*) is based on the neighborhood relations of the sensor defined by the 3D Voronoi diagram as well as the configuration of the environment and the presence of the potential obstacles. The direction of movement is towards the farthest vertex of its Voronoi cell. The amount of the movement is defined by *initial_step_size* which is 80% of the vector’s length from the sensor to the farthest vertex.

Following the initialization of the 3D Voronoi data structure and the *PQ*, the iterative optimization process is defined using a while loop (Algorithm 1). In each iteration, we move the sensor on the top of the *PQ* towards the farthest vertex of its Voronoi polyhedron. It should be noted that for each sensor, the motion vector needs to be projected on the nearest deployable surface. This is needed to make sure that the sensor has a support to be installed in the 3D environment. Then, the selected sensor is moved in this direction towards its new deployable position. The step size is decreased proportional to the maximum number of iterations which can be defined by the user (e.g., *Iteration* = 1000). Indeed, the step size is decreased to create a trade-off between exploration and exploitation in the search space. This ensures the avoidance of divergence in the optimization process results [33]. The sensor position with the highest gain coverage is updated in the while loop and the 3D Voronoi diagram is updated to reflect the new configuration of the network. Next, the updated coverage gains for the moved sensor (*g’_S’u_*) and its neighbors (*g’_NSj_*) are added in the *PQ* and sorted based on the highest coverage gain among the sensor. In the case of the presence of a permanent obstacle in the sensor movement direction, the sensor needs to be kept away from the obstacle. For this purpose, we define a distance constraint to avoid the obstruction of the sensor’s field of view. The while loop is stagnated with the terminated condition when the maximum coverage gain is less than a predefined coverage gain threshold (*ε*) for 10 iterations in a row.

## 5. Case Study

### 5.1. Model of the Indoor Environment and Sensors

For this research, Quebec City Convention Center was selected as the study area. This building hosts many national and international events and conferences. Sensor deployment with the goal of optimal coverage inside this building is a complex task due to its complexities of structure. This four-story building includes many conference rooms and corridors as well as several entrances, escalators, and elevators. This represents many cells and surfaces (walls, ceilings, floors, and obstacles) with complex architectural configurations and designs for the same floor; furthermore, the heights of the ceilings are not uniformly designed, which makes sensor deployment more difficult. Geometrically, the spaces of this building are discrete and non-convex. Traditionally, sensor deployment in such a building is carried out with a trial-and-error method, which usually results in several gaps in the network coverage. In addition, the presence of obstacles such as columns and furniture inside the building complexifies the deployment process and hence is generally neglected. Although the study area is a multi-floor building, for the sake of simplicity and feasibility we only considered the 4th floor as the test area for this study. More specifically, the study area included two main corridors and the main hall (generally used for exhibition booths, poster installations, or as a dining area for participants).

**Figure 7:**
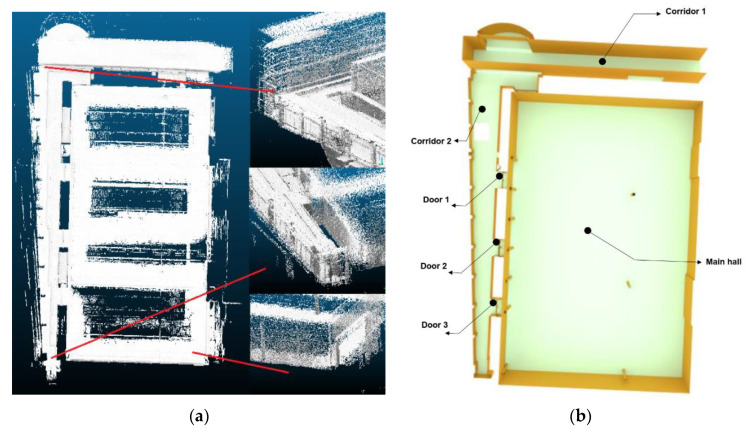
Quebec Convention Center building, (**a**) 3D point cloud, and (**b**) 3D vector model.

To be able to integrate the indoor 3D model of this building with our proposed deployment method, we first acquired a set of LiDAR 3D point clouds using a GeoSLAM device. This device is designed mainly for indoor environment scanning (Figure 7a). Then, we semi-automatically converted the obtained 3D point clouds into 3D vector model using the Blender software (Figure 7b). This model was composed of a set of surfaces in a 3D space. Using the Google SketchUp software, we semantically classified the 3D surfaces into walls, ceilings, floors, and obstacles. Finally, with the IndoorGML standard, we converted the classified 3D vector model into the IndoorGML model. In the next step, we extracted the information on the 3D indoor environment from this model for our algorithm based on the cell and geometry identifier. At the end, all surfaces composing our 3D environment were extracted with geometric information, such as coordinates of the surface limits, normal vectors, and semantic information including floor numbers, cell types (e.g., corridor, hall, and room), and the type of surface (e.g., walls, ceilings, floors, and obstacles). This information actually helped us in defining the sensor deployable positions as well as for the estimation of the sensor network coverage (specifying the covered area) for the defined environment.

**Figure 8:**
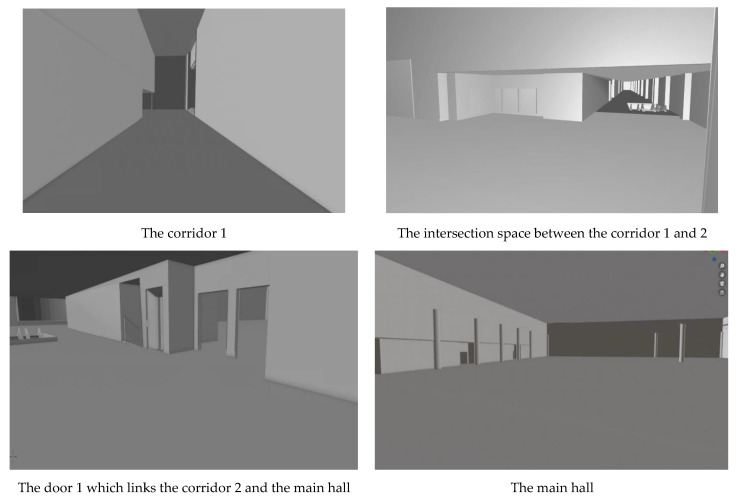
Indoor views of different spaces (corridors and the main hall) which show the complexity of surfaces in the Convention Center building.

The 3D model of the indoor environment selected for this experiment was composed of about 1000 surfaces, including walls and ceilings on which the sensors were deployed. As depicted in the Figure 8, the management of these surfaces as well as their topological relations in 3D space is much more complex compared to a simple 2D representation of the environment. Sensor deployment cannot be easily accomplished in such an environment because of the differences in the heights of ceilings and complexities of topology between the walls and ceiling surfaces (Figure 8). In addition, there were many obstacles that complicate sensor placement and the optimization process. In our case study, the 3D environment included nine columns in the main hall. These columns were modeled as cylinders that were themselves composed of many smaller surfaces. In addition, several non-convex surfaces had to be managed inside the corridors and the main hall. These surfaces created a large search space for the computation of maximum coverage for the optimization process. Another time-consuming aspect was related to the use of the vector-based model for the computation of coverage. Vector-based coverage computation demands more computation effort; however, it results in more accurate coverage estimation. Raster or voxel-based models may be simpler, but they may be less accurate for coverage estimation due to their limited resolutions.

To implement the proposed sensor placement optimization algorithm, as a first experiment, we considered omnidirectional cameras without the limitation of the viewing distance and field angle. We defined the mathematical model of these cameras as a hemisphere with an infinite radius. Information in an indoor environment, such as walls and obstacles, limits the range of the sensing area and reduces the covered areas by these cameras. We selected four cameras for the deployment implementation. These cameras could only be placed on ceilings and walls (part of the walls with a height constraint from the floors were considered for facilitating camera installation). The area of interest covered by these cameras was considered to be the floors. In addition, we designed two other experiments for sensitivity analysis regarding the number of sensors and sensing range. For these experiments, we increased the number of cameras and limited their sensing range.

### 5.2. Experimental Results and Discussions

**Figure 9:**
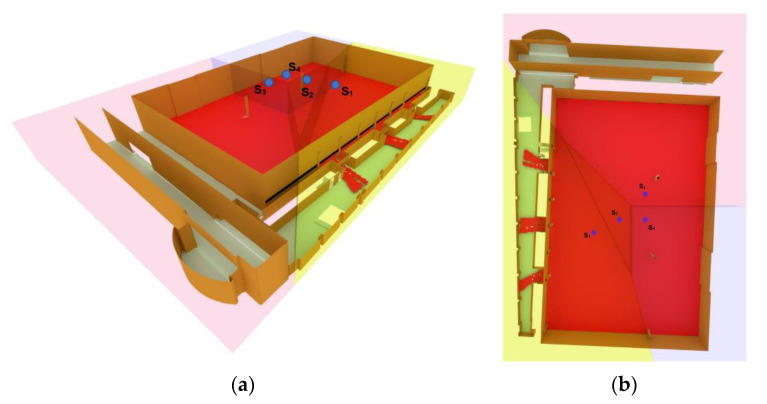
Initial position of cameras in the main hall (**a**) side view and (**b**) top view.

For the first experiment, the proposed 3D Voronoi algorithm was implemented to deploy four omnidirectional cameras with unlimited sensing range using the 3D vector model presented in the previous section. The deployable areas of the cameras were assumed to be on the ceiling and the walls with a distance of at least two meters from the floor. Furthermore, several columns in the main hall were considered as obstacles and their surfaces were identified in the model to consider their presence during the sensor deployment optimization process. For the initialization step, four cameras were randomly positioned on the central ceiling of the main hall (Figure 9).

**Figure 10:**
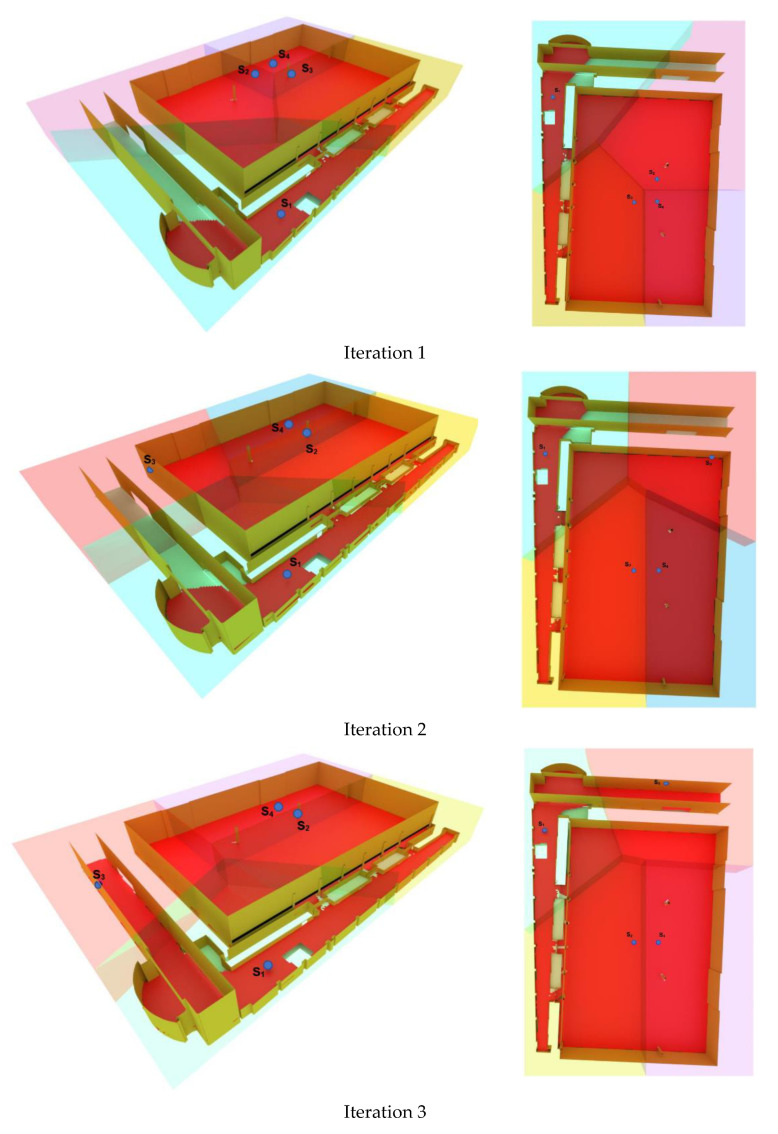

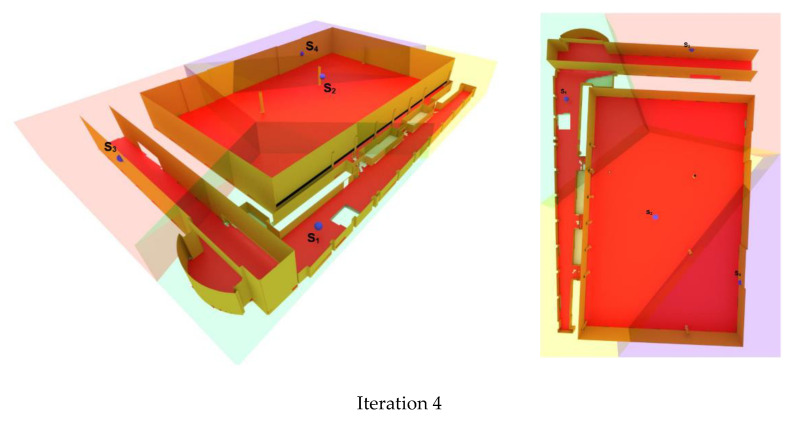
The result of the proposed 3D Voronoi approach including the positions of sensors (blue points), and their coverage (red area) for the first four iterations.

Then, at each iteration, according to the algorithm’s process, the cameras moved along the farthest points of each corresponding Voronoi cell, with the projection of their locations to the nearest deployable surface. At each iteration, the camera with the most important contribution to the improvement of global coverage was chosen to be moved to its new position. Figure 10 shows the location of the cameras for four subsequent iterations. In this figure, the colored-transparent areas show the 3D Voronoi cells of the cameras. The red area indicates the area covered by the cameras and the blue points indicate the location of the cameras.

**Figure 11:**
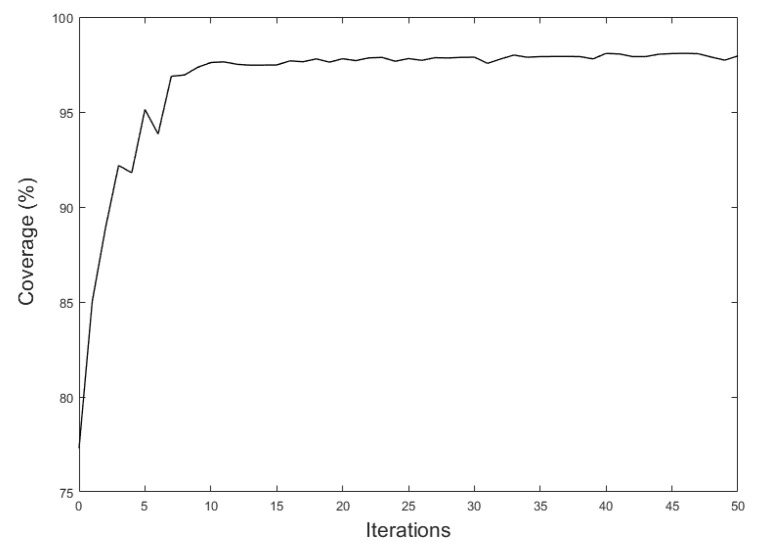
Camera coverage convergence diagram for the 3D Voronoi approach in 50 iterations.

**Figure 12:**
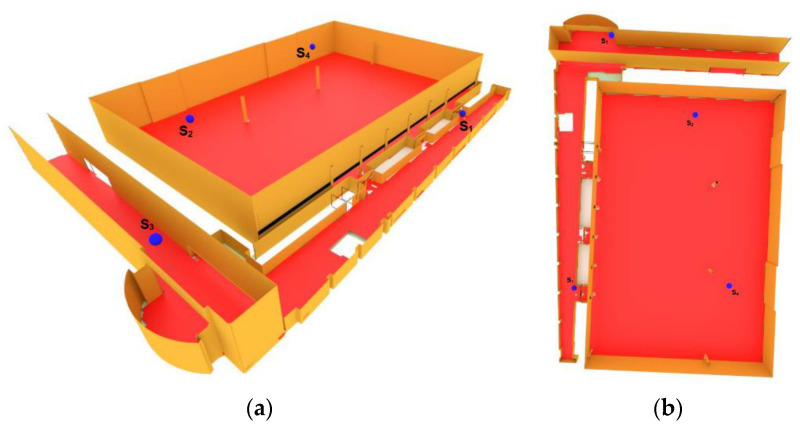
Final locations of cameras obtained by the 3D Voronoi approach are shown in (**a**) side view and (**b**) top view.

With 50 iterations, the coverage of the camera network was improved to 98% of the area. Figure 11 shows the convergence diagram of the 3D Voronoi approach for this case study after 50 iterations. As can be seen, the camera network coverage after 20 iterations reached the converged state with an optimal coverage. Figure 12 illustrates the final configuration of cameras with optimal coverage. This shows that our proposed method could deploy the sensors not only on the ceilings but also on the walls to reach the optimal coverage.

### 5.3. Comparison and Validation

To show that our proposed algorithm reached an optimal coverage, we compared our algorithm with the Genetic Algorithm (GA) and with the Covariance Matrix Adaptation Evolution Strategy (CMA-ES) as two examples of global methods [34]. To implement the GA and CMA-ES for the deployment of the same set of cameras on the ceilings or walls, it was necessary to prepare the algorithms for the optimization process. For this purpose, we first randomly generated the initial solutions for camera configurations (solution (*i*) = {*X1*, *X2*, *X3*, *X4*, *Y1*, *Y2*, *Y3*, *Y4*, *Z1*, *Z2*, *Z3*, *Z4*}) within the 3D bounding box of the environment. Then, to achieve the deployable position of the cameras, the cameras were projected on the nearest wall or ceiling surface so that we could find the right solution to calculate their coverage. In the next steps of the algorithms, the solutions were also projected on the nearby surfaces.

The initial parameters of the GA including population size, offspring percentage, and mutation percentage were selected with a trial and error method to 100%, 70%, and 20%, respectively. In addition, the setting parameters of CMA-ES are population (*λ*), formation of solutions means, and direction (*σ*) that were considered as 3 + 4 × [*ln(n)*] (*n* = 3 × (the number of sensors)), λ/2, and 0.167, respectively.

**Figure 13:**
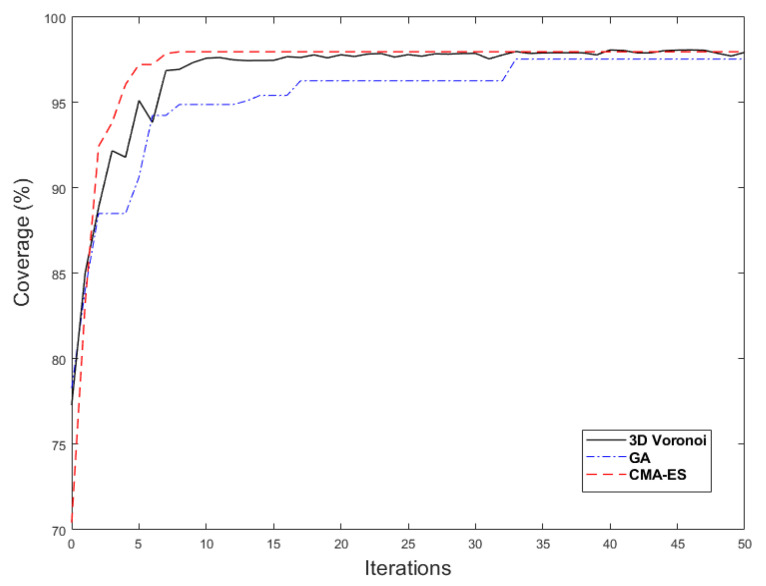
Convergence diagrams of 3D Voronoi, GA, and CMA-ES.

To eliminate the random errors, the proposed 3D Voronoi approach, as well as the GA and CMA-ES, were executed a hundred times. As seen in Figure 13, the 3D Voronoi approach converged quickly to the optimal value. In addition, the final optimal coverage value of the 3D Voronoi approach was greater than the coverage values of the GA and CMA-ES algorithms.

According to Table 1, it can be concluded that the computational time of the 3D Voronoi approach was six times less than the computational time of the GA and CMA-ES algorithms. Moreover, the standard deviation values reported in Table 1 indicate that the final coverage level of sensors in the 3D Voronoi algorithm was more robust than GA and CMA-ES algorithms. This is because our approach based on a 3D Voronoi diagram is more sensitive to the spatial information from the environment and sensor configuration. This outcome of incorporating the physical information in the 3D algorithm makes the sensor network deployment coverage improve over the physical space.

### 5.4. Sensitivity and Efficiency Analysis

To further evaluate the efficiency of the proposed 3D Voronoi approach with respect to the sensing range and the number of cameras, we implemented several experiments and present the obtained results in Table 2 and Table 3. First, the comparison of algorithms was carried out for each experiment with different sensing ranges including 30, 50, 70, and 90 m for deploying four cameras with an equal sensing range on the fourth floor of the Convention Center building to assess the sensitivity of algorithms (3D Voronoi, GA, and CMA-ES) to the sensing range of the cameras. Second, the selected number of cameras was, respectively, 4, 6, 8, and 10 for each experiment to assess the efficiency of algorithms based on the number of cameras. The average best coverage values, standard deviations, and computation time were computed by the algorithms for each experiment. It should be mentioned that we fixed the sensing range for assessing the number of sensors and vice versa.

Table 2 shows the obtained results for the three algorithms for deploying four cameras with different sensing ranges. The performance time for all of the experiments indicated that the lowest computation cost belonged to the 3D Voronoi-based algorithm compared to the GA and CMA-ES algorithms. Concerning the sensing range, the robustness was augmented by increasing the sensing range in each experiment. For higher sensing ranges, the algorithms performed better to reach higher optimum due to the efficiency of camera sensing, except for GA where the coverage was slightly decreased for a sensing range of 90 m.

Regarding the number of cameras, several experiments were carried out for the deployment of 4, 6, 8, and 10 cameras using the three mentioned algorithms. The results of these experiments are shown in Table 3.

Table 3 indicates that when the number of cameras increased, the robustness (based on the standard variation in 100 runs (column 4)) of the algorithms also increased. However, the robustness of GA and CMA-ES algorithms was lower than the proposed 3D Voronoi-based algorithm. This is because the proposed algorithm better considers the spatial structure of the environment. Increasing the number of cameras increased the coverage value in all algorithms. Although the coverage value was improved for 3D Voronoi and CMA-ES, there was an exception for GA in that it had higher coverage with 8 cameras than with 10 cameras. We presume that this occurs because of the number of parameter settings and the population size of GA, which are higher compared to the other algorithms. The results show that our algorithm has better improved the coverage values when the number of cameras was more than six cameras compared to GA and CMA-ES. Finally, the results shown in Table 3 reveal that the computation costs of the algorithms were higher by the increase in the number of cameras.

## 6. Conclusions and Future Works

Optimal deployment of a sensor network within a 3D indoor environment is a challenging task. Traditionally, sensor placement in such environments is done using a trial-and-error method. More recently, a few optimization algorithms have been proposed for the optimal deployment of sensor networks in indoor environments. However, these algorithms generally oversimplify the environment complexities and are mostly developed for 2D environments. In addition, they rarely consider the presence of obstacles embedded in the environment. Furthermore, in these approaches, sensing models of individual sensors are also oversimplified. Our aim in this research was to propose a new algorithm for the optimization of a sensor network in a 3D indoor environment that allows better consideration of the spatial characteristics of the environment to support applications such as mobility and security surveillance.

Here, we proposed a new local optimization algorithm based on a 3D Voronoi diagram for optimal sensor deployment in an indoor environment. The proposed optimization algorithm integrates the IndoorGML model as well as a 3D Voronoi diagram for the representation of a 3D indoor environment as well as the distribution of the sensors and their neighborhood relations respectively. The proposed solution includes a local optimization algorithm that benefits from the 3D Voronoi data structure to define and manage sensor movement for optimization purposes. It also allows better consideration of the presence of obstacles (e.g., columns).

For the validation of the proposed algorithm, it was implemented and tested for three sensor placement scenarios (camera network) in the Quebec Convention Center. The results show that the camera network coverage reached almost 98% from the initial random state using the 3D Voronoi approach. We also compared this method with the GA and CMA-ES algorithms to evaluate the performance of this method and showed that the final coverage value tends to an optimum value and is comparable with the results obtained from GA and CMA-ES algorithms. The algorithm tended to perform even better when the number of cameras is higher than eight for this experiment for the selected 3D indoor environment. The results also reveal that for this case study, our proposed algorithm was more than six times more efficient than the global optimization algorithms and achieved optimal coverage with lower computational time.

For future research, we seek to extend the capacity of the algorithm for the deployment of a multi-sensor network in an indoor environment including cameras and positioning and tracking sensors for different applications related to mobility, security, and evacuation. In addition to the environment and sensor network constraints, we will further integrate application-related constraints in the optimization of the sensor network. For instance, prioritizing sensor placement in areas where people more likely encounter problems in their wayfinding and in their mobility tasks.

## Figures and Tables

**Table 1:** Results of 3D Voronoi, GA, and CMA-ES algorithms including coverage average (AVG-coverage) and its standard deviation (SD-coverage).

Method	AVG-Coverage (%)	SD-Coverage (%)	Time (s)
3D Voronoi	98.86	0.76	646.45
GA	98.1	1.64	3898.86
CMA-ES	98.45	1.82	3264.92

**Table 2:** Results of 3D Voronoi, GA, and CMA-ES algorithms for four cameras.

Method	Sensing Range	AVG-Coverage (%)	SD-Coverage (%)	Time (s)
3D Voronoi	30	67.44	5.69	176.62
	50	96.17	1.94	304.92
	70	98.16	1.16	486.503
	90	98.61	0.88	654.734
GA	30	70.42	6.74	2112.72
	50	93.50	2.76	2663.72
	70	98.19	2.32	3647.88
	90	97.55	1.56	3820.56
CMA-ES	30	70.74	5.98	2089.38
	50	94.68	2.20	2509.52
	70	98.08	1.60	3511.1
	90	98.52	1.21	3858.34

**Table 3:** Results of 3D Voronoi, GA, and CMA-ES algorithms for different numbers of cameras with 30 m sensing range.

Method	Number of Cameras	AVG-Coverage (%)	SD-Coverage (%)	Time (s)
3D Voronoi	4	67.44	5.69	176.62
	6	86.48	5.07	263.02
	8	95.42	2.01	369.82
	10	98.08	1.90	494.09
GA	4	70.42	6.74	2112.72
	6	78.93	6.32	3431.64
	8	89.68	4.24	4114.16
	10	86.70	2.89	6726.16
CMA-ES	4	70.74	5.98	2089.38
	6	86.06	6.12	3252.98
	8	86.48	3.45	4434.94
	10	90.63	2.37	5867.38

## Data Availability

Not applicable.
